# Derived hematological indices during peripheral blood stem cell mobilization in the plerixafor era: insightful dynamics but limited predictive value

**DOI:** 10.1016/j.htct.2026.106474

**Published:** 2026-06-02

**Authors:** Mohandoss Murugesan, Lakshmi P. Niveditha, Edakkadath Raghavan Sindhu, Chandran K. Nair

**Affiliations:** a Transfusion Medicine, Malabar Cancer Centre (Postgraduate Institute of Oncology Sciences and Research), Thalassery, Kerala, India; b Project Resident, Clinical Lab Services, Malabar Cancer Centre (Postgraduate Institute of Oncology Sciences and Research), Thalassery, Kerala, India; c Biochemistry, Malabar Cancer Centre (Postgraduate Institute of Oncology Sciences and Research), Thalassery, Kerala, India; d Clinical Hematology, Malabar Cancer Centre, Thalassery, Kerala, India

**Keywords:** Peripheral blood stem cells, Plerixafor, Granulocyte colony-stimulating factor, Derived hematological indexes, CD34^+^ cell count

## Abstract

**Introduction:**

Derived hematological indices reflect immune activation, but their dynamics during the plerixafor era and their predictive value for stem cell yield remain unclear.

**Objectives:**

To assess changes in derived hematological inflammatory markers during peripheral blood stem cell mobilization in autologous patients and allogeneic donors, and to evaluate their correlation with CD34^+^ stem cell yield. These markers include ratios (neutrophil-to-lymphocyte, platelet-to-lymphocyte and lymphocyte-to-monocyte), and composite indices (systemic immune-inflammation and systemic inflammation response indices).

**Methods:**

A retrospective analysis was conducted of 127 autologous and 53 allogeneic mobilization events between January 2019 and March 2024. Blood counts and derived indices were analyzed before and after granulocyte colony-stimulating factor administration with and without plerixafor. CD34^+^ cell enumeration was performed after mobilization. Dynamic changes in inflammatory indices and their correlation with CD34^+^ counts were assessed.

**Results:**

After mobilization, there was a significant increase in the neutrophil-to-lymphocyte ratio, systemic immune-inflammation index, and systemic inflammation response index, and a decrease in the platelet-to-lymphocyte and lymphocyte-to-monocyte ratios in both groups. While allogeneic donors had higher CD34^+^ counts (median 110 cells/µL versus 48 cells/µL in autologous), no consistent correlation was found between dynamic inflammatory marker changes and CD34^+^ yield. Plerixafor use in autologous patients significantly influenced the platelet-to-lymphocyte ratio and systemic immune-inflammation index dynamics, highlighting its role in modulating the acute inflammatory response in the plerixafor era.

**Conclusions:**

Inflammatory markers change significantly during mobilization with granulocyte colony-stimulating factor, reflecting acute immune activation. However, the studied markers do not predict CD34^+^ yield and should not replace direct enumeration. Their role may be better suited to complement biological understanding rather than clinical decision-making.

## Introduction

Peripheral blood stem cell (PBSC) mobilization induces significant hematological changes, including elevated white blood cell (WBC) counts, increased CD34^+^ cell counts, and occasional thrombocytopenia [1, 2, 3]. These shifts reflect the physiological response to mobilization and the systemic demand for hematopoietic progenitor cells. The introduction of agents such as plerixafor, which antagonizes the CXCR4-SDF1α axis, has further improved mobilization efficiency, particularly in patients who respond poorly to granulocyte colony-stimulating factor (G-CSF) alone [4].

Pre-apheresis peripheral blood CD34^+^ cell enumeration remains the gold standard for predicting stem cell yield and guiding collection timing [1]. However, this technique requires flow cytometry, which can be time-consuming and resource-intensive. Conversely, simple, cost-effective markers derived from routine complete blood counts, such as the Neutrophil-to-Lymphocyte Ratio (NLR), Platelet-to-Lymphocyte Ratio (PLR), Lymphocyte-to-Monocyte Ratio (LMR), and composite indices like the Systemic Immune-Inflammation Index (SII) and Systemic Inflammation Response Index (SIRI), have gained attention as surrogate markers of inflammation and immune activation [5, 6, 7]. These indices have been used in cancer settings to assess prognosis, treatment response, and systemic inflammation however, their potential predictive value for stem cell yield remains unclear [8, 9, 10].

G-CSF–mediated mobilization is known to provoke a transient inflammatory response, which may be reflected in these derived markers [11]. While historical data on hematological indices exists for G-CSF mobilization, there is a scarcity of data regarding their behavior in the current plerixafor era. Plerixafor’s unique mechanism, antagonizing the CXCR4-SDF1α axis, may alter the systemic inflammatory milieu differently to G-CSF alone. Understanding the dynamics could enhance our ability to monitor and optimize mobilization strategies without relying solely on CD34^+^ enumeration. This study addresses this gap by including a significant proportion of patients requiring plerixafor.

This study evaluates changes in derived hematological inflammatory indices during PBSC mobilization in both autologous patients and allogeneic donors. It also investigates whether these markers correlate with CD34^+^ cell counts and could serve as alternative indicators of mobilization adequacy.

## Methods

This study was a retrospective observational analysis conducted at a tertiary care center, assessing autologous patients and allogeneic donors who underwent PBSC mobilization followed by apheresis for hematopoietic stem cell transplantation (HSCT). The data collection period spanned from January 2019 to April 2024. The study was approved by the Institutional Review Board.

The study population included autologous patients undergoing mobilization as part of treatment for hematologic malignancies (e.g., lymphoma, multiple myeloma) and allogeneic donors, typically related healthy individuals, undergoing mobilization for matched or haploidentical recipient transplantation.

The inclusion criteria were autologous patients or allogeneic donors who received G-CSF-based mobilization, with or without plerixafor. Those with incomplete pre- and post-mobilization hematological and CD34^+^ cell count data were excluded.

### Data collection

Clinical and laboratory data were extracted from hospital medical records, including patient files and donor evaluation charts. Apheresis unit records, which included mobilization regimen details and stem cell product characteristics, were collected.

The following variables were collected and entered into a standardized proforma:

• Demographic details: Age, sex.
• Mobilization regimen: Number of G-CSF doses, use and number of doses of plerixafor.
• Laboratory parameters:
  • Hemoglobin, hematocrit, total white blood cell (WBC) count, percentage of neutrophils, lymphocytes, monocyte and platelet count, recorded before and after mobilization.
  • Peripheral blood CD34^+^ counts (pre-apheresis) measured by flow cytometry.

### ***Calculation of derived inflammatory indices*** [6,8,12,13]

Derived hematological inflammatory markers were calculated from complete blood count (CBC) values as follows:

• Neutrophil-to-Lymphocyte Ratio (NLR) = Absolute neutrophil count / Absolute lymphocyte count
• Platelet-to-Lymphocyte Ratio (PLR) = Platelet count / Absolute lymphocyte count
• Lymphocyte-to-Monocyte Ratio (LMR) = Absolute lymphocyte count / Monocyte count
• Systemic Immune-Inflammation Index (SII) = (Neutrophil count × Platelet count) / Lymphocyte count
• Systemic Inflammation Response Index (SIRI) = (Neutrophil count × Monocyte count) / Lymphocyte count

For each index, values were calculated both before and after mobilization, and dynamic changes (Δ values) were computed as post-mobilization minus pre-mobilization values. Also to analyze the magnitude of change, mobilization signatures of these indexes were estimated by post-value / pre-value between autologous patients and allogeneic donors who received plerixafor or not.

### Statistical analysis

Data were analyzed using statistical software. Continuous variables were expressed as median with range. Comparisons of pre- and post-mobilization values were performed using paired statistical tests (e.g., Wilcoxon signed-rank test or paired *t*-test, depending on distribution). Differences between autologous and allogeneic groups were assessed using the Mann–Whitney U test or independent *t*-tests. Correlations between derived inflammatory indices and CD34^+^ counts were assessed using Spearman’s rank correlation coefficient. Receiver Operating Characteristic (ROC) curves were plotted to evaluate the predictive ability of dynamic indices for achieving CD34^+^ counts >50 cells/µL. A p-value <0.05 was considered statistically significant.

## Results

Reports of autologous patients and allogeneic donors who underwent stem cell mobilization and harvesting for transplantation were consecutively analyzed during the period from January 2019 to March 2024. The number of events taken for analysis in the autologous setting was 127 while it was 53 events for allogenic donors.

The median ages in the autologous and allogenic settings were 47 years and 39 years, respectively. Patients received G-CSF mobilization alone in 69.3 % of the events and plerixafor along with G-CSF in the remaining 30.7 %. While donors received G-CSF mobilization in 98.1 % and only 1 (1.9 %) donor required plerixafor along with G-CSF for mobilization. The number of days that G-CSF was administered is shown in Table 1.

**Table 1:** Demographics and mobilization regimen used in autologous patients and allogenic Donors.

	Autologous (*n* = 127)	Allogenic (*n* = 53)
	Frequency	Frequency
Age (Years) - Median (Range)	47 (4–65)	39 (2–69)
Gender – n ( %)		
Male	80 (63)	29 (54.7)
Female	47 (37)	24 (45.3)
G-CSF (days) – n ( %)		
5	84 (66.2)	49 (92.5)
6	36 (28.3)	3 (5.7)
7	6 (4.7)	0 (0)
8	1 (0.8)	1 (1.9)
Plerixafor (dose) – n ( %)		
None	88 (69.3)	52 (98.1)
1	35 (27.6)	1 (1.9)
2	4 (3.1)	-

G-CSF: granulocyte colony-stimulating factor.

Table 2 shows the baseline blood counts of both autologous patients and allogenic donors before and after the administration of G-CSF. There was a significant increase in the WBC count after mobilization in both groups. Additionally, lymphocyte percentages dropped, while neutrophil percentages rose sharply, indicating a shift in the immune cell profile. After mobilization the median CD34^+^ counts in autologous patients was 48 cells/µL whereas it was 110 cells/µL in allogenic donors.

**Table 2:** Blood Picture of autologous patients and allogeneic donors before and after mobilization with granulocyte colony-stimulating factor (G-CSF) (Median with range).

	Autologous Patients		Allogenic Donors	
	Before	After		After
**Hemoglobin** (g/dL)	11.9 (7.7–15.6)	11.7 (7.8–14.9)	13.1 (8.5–16.2)	13.3 (8.3–16.1)
**Hematocrit** ( %)	36.7 (23.4–46.9)	35.9 (24.9–44.5)	40.7 (26.6–47.9)	40.5 (27–48.5)
**WBC Count** (x 10^3^/µL)	6.60 (1.41–4.60)	42.10 (13.70–96.20)	7.69 (4.90–10.50)	43.20 (18.60–82.86)
**Platelet Count** (x10³/µL)	256 (71–769)	178 (48–511)	264 (155–442)	218 (125–405)
**Neutrophils** ( %)	60 (6–90)	84.2 (5.3–95)	52.1 (24.5–76)	83.5 (8.6–87.9)
**Lymphocytes** ( %)	28 (7–64)	7 (3–35)	37.3 (19.9–59)	9.2 (5.2–18)
**Monocytes** ( %)	8 (2.5–83.5)	5.25 (1–84)	6 (2–12.4)	6 (2– 0.8)
**CD34^+^ cells** (cells/µL)	Not done	48 (2–316)	Not done	110 (34–486)

WBC: White blood cell.

Table 3 shows the CD34 enumeration details by flow cytometry. Regarding mobilization efficacy, 11.3 % of autologous patients failed to reach the minimum threshold (<20 cells/µL), whereas all allogeneic donors exceeded this level. Furthermore, suboptimal mobilization (<50 cells/µL) was observed in 52.4 % of autologous patients, while the vast majority of allogeneic donors (92.5 %) achieved optimal counts (>50 cells/µL).

**Table 3:** Flow cytometric enumeration of peripheral blood CD34^+^ counts in autologous patients and allogenic donors.

		Autologous		Allogenic	
		Frequency	Percent	Frequency	Percent
**CD34^+^ Count**	**<20** cells/µL	14	11.3	0	0
	**>20** cells/µL	110	88.7	51	100
**CD34^+^ Count**	**<50** cells/µL	65	52.4	2	7.5
	**>50** cells/µL	59	47.6	49	92.5

In autologous donors, the NLR increased significantly from a normal baseline of 2.1 to 11.7 post-mobilization. Conversely, LMR, which was low at baseline (3.2), further declined to 1.3. While PLR was high before mobilization (148.8), it dropped sharply to 57.9. The SII increased from 0.565 to 1.925, and the SIRI rose from 1201.7 to 30,102.5, reflecting a robust systemic inflammatory response during the mobilization process.

**Figure 1:**
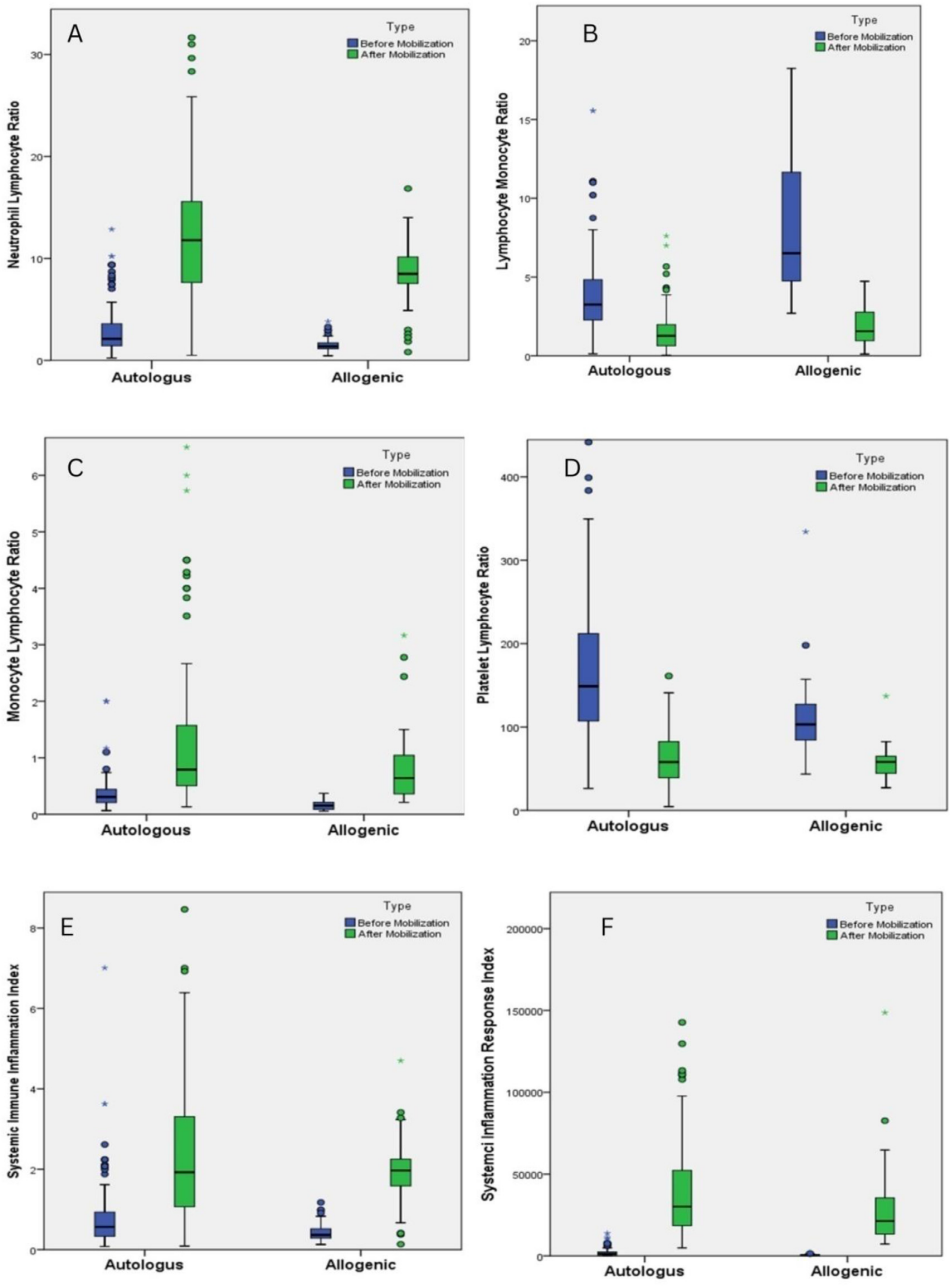
Changes in Derived Blood Indices before and after mobilization in Autologous patients and allogenic donors.

Similar trends were observed in autologous patients, characterized by a sharp increase in NLR (1.4 versus 8.5) and SIRI (564.0 versus 21,309.6), alongside a decrease in PLR (103.1 versus 58.1) and LMR (6.5 versus 1.6). Figure 1 provides a visual representation of the shifts in all derived inflammatory markers following mobilization.

Table 4 summarizes the dynamic changes in derived inflammatory markers following mobilization in both autologous patients and allogeneic donors. While dynamic changes in NLR and SII were observed in both groups, the differences were not statistically significant. However, significant differences between autologous and allogeneic cohorts were identified for the LMR (p-value = 0.003), MLR (p-value = 0.027), PLR (p-value = 0.006), and SIRI (p-value = 0.030), indicating that these specific markers may reflect differential inflammatory or hematologic responses to mobilization in the two populations.

**Table 4:** Dynamic Changes in Inflammatory markers with mobilization in autologous patients and allogenic donors.

	Autologous		Allogenic		p-value
	Median	Range	Median	Range	
**Dynamic NLR**	4.144	−1.0 to 30.8	4.845	−1.0 to 14.1	0.786
**Dynamic LMR**	−0.639	−1.0 to 12.4	−0.787	−1.0 to 0.5	**0.003**
**Dynamic PLR**	−0.612	−1.0 to 1.8	−0.480	−1.0 to 0.4	**0.006**
**Dynamic SII**	2.363	−1.0 to 22.1	3.619	−1.0 to 10.8	0.285
**Dynamic SIRI**	26.163	0 to 530.5	40.531	0 to 118.3	**0.030**

NLR: Neutrophil-to-Lymphocyte Ratio; LMR: Lymphocyte-to-Monocyte Ratio; PLR: Platelet-to-Lymphocyte Ratio; SII: Systemic Immune-Inflammation Index; SIRI: Systemic Inflammation Response Index.

Table 5 summarizes the impact of plerixafor use on inflammatory indices in autologous patients undergoing PBSC mobilization. At baseline (prior to mobilization), there were no statistically significant differences in any of the inflammatory markers between patients who received G-CSF alone and those who received G-CSF with plerixafor. However, post-mobilization, patients who received plerixafor showed significantly lower PLR (p-value < 0.01) and SII (p-value = 0.005) compared to those mobilized with G-CSF alone, suggesting a modulation of the inflammatory response by plerixafor. Further analysis of dynamic changes (Δ values) in these indices revealed that PLR (p-value < 0.01) and SIRI (p-value = 0.03) were significantly different between the two groups. No significant differences were observed in the dynamic changes of NLR, LMR, MLR, or SII. These findings suggest that while plerixafor does not influence baseline inflammatory status, it may attenuate specific inflammatory responses after mobilization, particularly for platelet- and monocyte-associated indices.

**Table 5:** Changes in blood indexes with plerixafor in autologous patients [median (range)].

Inflammatory Variable	Before Mobilization			After Mobilization		
	No Plerixafor	With Plerixafor	p-value	No Plerixafor	With Plerixafor	p-value
**NLR**	2.14 (0.2–12.9)	2.107 (0.6–10.2)	0.496	12.31 (0.5–31.7)	9.42 (0.9– 29.6)	0.241
**LMR**	3.03 (0.5–11.1)	3.83 (0.1–15.6)	0.181	1.31 (0.1–7.6)	1.17 (0.5–5.7)	0.52
**PLR**	148.85 (26.3–685.2)	148.85 (26.3–685.2)	0.987	62.09 (12.9–140.9)	40.20 (4.5–161.1)	**<0.01**
**SII index (x 10⁹/L)**	0.57 (0.1–7.0)	0.56 (0.1–3.6)	0.368	2.33 (0.1–8.5)	1.59 (0.2–5.0)	**0.005**
**SIRI index (x 10⁹/L)**	1294.22 (159.4–13,802)	1116.78 (135–10,354)	0.143	27,398.25 (4862–267,904)	34,633.86 (6524–470,854)	0.461

NLR: Neutrophil-to-Lymphocyte Ratio; LMR: Lymphocyte-to-Monocyte Ratio; PLR: Platelet-to-Lymphocyte Ratio; SII: Systemic Immune-Inflammation Index; SIRI: Systemic Inflammation Response Index.

**Figure 2:**
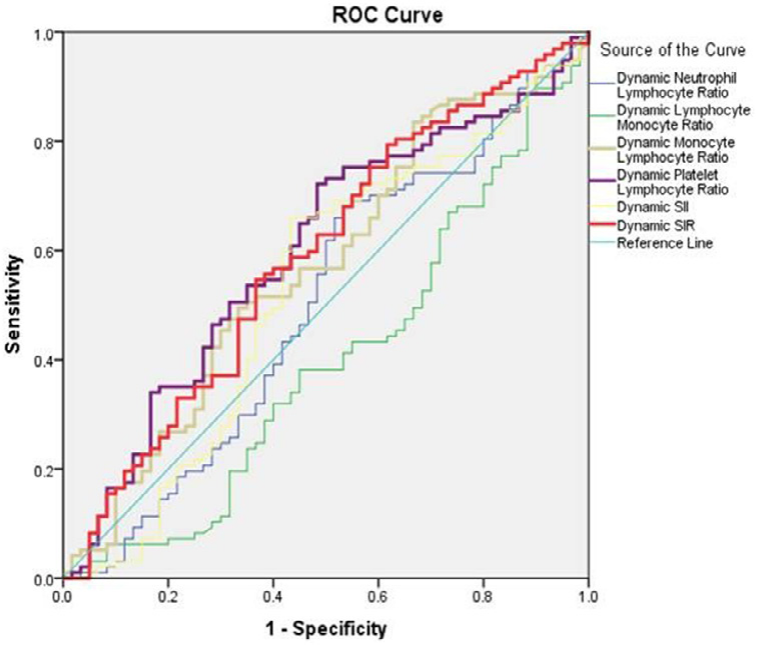
Receiver Operating Characteristics curve demonstrating dynamic changes in blood indices comparing before and after mobilization in autologous patients and allogenic donors with peripheral blood CD34^+^ Count >50 cells/µL.

No significant correlation was found between peripheral blood CD34^+^ cell counts and the dynamic changes in derived variables. Consequently, ROC analysis (Figure 2) demonstrated low predictive power, with AUC values ranging between 0.5 and 0.6.

Table 6 captures the dynamic shift in inflammatory markers, highlighting how plerixafor significantly blunts the velocity of change of specific indices. Subdividing the autologous cohort based on plerixafor administration demonstrated that the drug significantly alters PLR kinetics (p-value < 0.01).

**Table 6:** Mobilization signature ratios (post-mobilization/pre-mobilization velocity) in autologous patients mobilized with and without plerixafor.

Inflammatory Variable	Plerixafor		p-value
	No (*n* = 92)	Yes (*n* = 35)	
**NLR**	5.32 (0.34–31.81)	5.38 (0.35–18.39)	0.840
**LMR**	0.39 (0.03–2.00)	0.29 (0.02–13.42)	0.158
**PLR**	0.46 (0.09–2.76)	0.25 (0.04–1.31)	**<0.01**
**SII**	3.91 (0.22–14.16)	2.34 (0.19–23.08)	0.070
**SIRI**	25.80 (1.98–531.45)	33.75 (1.83–289.51)	0.158

NLR: Neutrophil-to-Lymphocyte Ratio; LMR: Lymphocyte-to-Monocyte Ratio; PLR: Platelet-to-Lymphocyte Ratio; SII: Systemic Immune-Inflammation Index; SIRI: Systemic Inflammation Response Index.

## Discussion

This study aimed to evaluate the dynamics of derived hematological inflammatory markers during PBSC mobilization in both autologous patients and allogeneic donors. In addition, it explored the correlation of these markers with CD34^+^ cell enumeration, which remains the gold standard for assessing mobilization efficacy and guiding apheresis timing.

G-CSF mobilization led to significant hematological shifts in both autologous patients and allogenic donors, including elevated total WBC and neutrophil counts, and relative lymphopenia. Consequently, derived inflammatory indices such as NLR, SII, and SIRI increased markedly, indicating a robust acute inflammatory response. In contrast, PLR and LMR decreased significantly after mobilization. These changes reflect the characteristic pro-inflammatory state induced by G-CSF and are consistent with earlier studies linking such indices to systemic inflammation and cancer prognosis [8,14, 15, 16].

The results revealed that while inflammatory markers change consistently in both autologous and allogeneic settings, some parameters, specifically LMR, MLR, PLR, and SIRI, differed significantly between the groups. These differences likely stem from the disease burden and exposure to prior treatment in autologous recipients, conditions that are absent in healthy allogeneic donors.

Notably, mobilization efficiency, as measured by CD34^+^ counts, was significantly higher in allogeneic donors (median = 110 cells/µL) compared to autologous patients (median = 48 cells/µL). Only one donor required plerixafor, while 27.6 % of autologous patients did. This highlights the greater mobilization challenges in the latter group.

In the present cohort, the use of plerixafor introduced a distinct inflammatory signature compared to mobilization with G-CSF alone. While baseline values were similar, the plerixafor subgroup exhibited significantly lower post-mobilization PLR (p-value < 0.01) and SII (p-value = 0.005). More importantly, the dynamic shifts for PLR (p-value < 0.01) and SIRI (p-value = 0.03) were significantly altered in these patients. These findings suggest that the addition of a CXCR4 antagonist does more than just enhance CD34^+^ egress; it may exert a unique modulatory effect on the systemic inflammatory milieu. By potentially attenuating G-CSF-induced responses in platelet and monocyte-associated indices, plerixafor appears to create a different kinetic profile, emphasizing the need to interpret these biomarkers differently in the modern plerixafor era of mobilization.

Interestingly, SII and SIRI showed the highest fold-increases after mobilization: 2-fold in autologous patients and between 5- and 37-fold in allogeneic donors. This disparity highlights a profound, albeit varying, systemic response to G-CSF across the two cohorts. While the magnitude of these shifts coincided with the superior mobilization observed in the allogeneic cohort (median 110 CD34^+^ cells/µL versus 48 cells/µL in autologous), the data of this study did not show a direct correlation between individual index increases and stem cell yield within the groups. Therefore, rather than serving as a direct measure of mobilization efficacy, these indices likely reflect the intensity of the acute inflammatory stimulus caused by the mobilization regimen. These findings contrast with their traditional prognostic role in oncology, where elevated baseline values typically correlate with poor clinical outcomes [14,15]. Future dose-effect analyses would be required to determine if the degree of index elevation can quantitatively predict yield.

LMR, previously associated with better survival in malignancy, decreased significantly (by 50–70 %) in both groups, indicating mobilization-associated lymphopenia and monocyte predominance [16]. This shift aligns with effective mobilization, although it diverges from survival data, again highlighting the distinct immune environment during stem cell collection.

Despite these significant changes, no dynamic marker showed a consistent correlation with CD34^+^ counts. ROC curve analyses yielded only marginally elevated area under curve (AUC) values for PLR, SIRI, and MLR and failed to establish any of these markers as reliable predictors for peripheral blood CD34^+^ cell counts above 50 cells/µL. This underscores the limited utility of inflammatory markers as standalone biomarkers in pre-apheresis decision-making. Enumeration of CD34^+^ cells by flow cytometry remains the most accurate, rapid, and actionable method for assessing mobilization success.

The mobilization signature ratio (the ratio of post- to pre-mobilization values) was analyzed to further explore the plerixafor era dynamics. The most striking finding was in the PLR, where the velocity of change was significantly lower in the plerixafor group (0.25 versus 0.46 in the G-CSF alone group; p-value < 0.01). This indicates a more pronounced suppression of PLR when plerixafor is added to the regimen.

Although prior studies have explored WBC counts, total nucleated cells, and even biochemical markers like CRP and albumin as surrogate predictors of stem cell yield, their utility remains suboptimal. [17].

A major strength of this analysis is its alignment with contemporary mobilization strategies. By analyzing a real-world cohort of autologous patients where plerixafor was utilized for suboptimal responders, the study provided a more comprehensive view of how modern pharmacological interventions influence these indices.

The findings of the present study align with this, suggesting that while derived hematological indices provide insight into the physiological milieu of mobilization, they lack specificity and predictive strength for clinical utility. Future research should explore composite scoring systems, approaches integrating inflammatory indices with clinical and laboratory parameters to improve predictive accuracy for mobilization success.

## Conclusion

PBSC mobilization with G-CSF, with or without plerixafor, is associated with pronounced shifts in derived hematological inflammatory markers, including significant increases in NLR, SII, and SIRI, and reductions in LMR and PLR. These changes reflect an acute systemic inflammatory response, characteristic of effective stem cell mobilization. Although the inflammatory indices vary between autologous and allogeneic settings and are influenced by mobilization regimens, particularly plerixafor use, they do not reliably predict CD34^+^ stem cell yield. The absence of correlation and low predictive accuracy of ROC analysis limits their application as standalone biomarkers. Flow cytometric enumeration of CD34^+^ cells remains the most robust and clinically meaningful measure for guiding stem cell collection.

## Financial disclosure

There was no funding utilized for this study.

## Data availability

The data that support the findings of this study are available from the corresponding author upon reasonable request.

## Conflicts of interest

There was no conflict of interest.
